# Alphavirus Infection: Host Cell Shut-Off and Inhibition of Antiviral Responses

**DOI:** 10.3390/v8060166

**Published:** 2016-06-11

**Authors:** Jelke J. Fros, Gorben P. Pijlman

**Affiliations:** 1 Nuffield Department of Medicine, Peter Medawar Building for Pathogen Research, University of Oxford, Oxford OX1 3SY, England, UK; jelke.fros@ndm.ox.ac.uk; 2 Laboratory of Virology, Wageningen University, Droevendaalsesteeg 1, 6708 PB, Wageningen 6700 AB, The Netherlands

**Keywords:** alphavirus, chikungunya, Sindbis, Semliki Forest, host shut-off, transcription, translation, antiviral response, stress granules, interferon, unfolded protein response

## Abstract

Alphaviruses cause debilitating disease in humans and animals and are transmitted by blood-feeding arthropods, typically mosquitoes. With a traditional focus on two models, Sindbis virus and Semliki Forest virus, alphavirus research has significantly intensified in the last decade partly due to the re-emergence and dramatic expansion of chikungunya virus in Asia, Europe, and the Americas. As a consequence, alphavirus–host interactions are now understood in much more molecular detail, and important novel mechanisms have been elucidated. It has become clear that alphaviruses not only cause a general host shut-off in infected vertebrate cells, but also specifically suppress different host antiviral pathways using their viral nonstructural proteins, nsP2 and nsP3. Here we review the current state of the art of alphavirus host cell shut-off of viral transcription and translation, and describe recent insights in viral subversion of interferon induction and signaling, the unfolded protein response, and stress granule assembly.

## 1. Introduction

The Alphavirus genus of the family *Togaviridae* contains about 30 viruses, most of which are transmitted between vertebrate hosts by mosquito vectors. Exceptions are Buggy Creek virus and Southern elephant seal virus, which are transmitted by swallow bugs and seal lice, respectively [1,2]. Unique members of the genus are Eilat virus, which is a mosquito-specific alphavirus [3], and the aquatic salmonid alphavirus (SAV), which can be transmitted from fish to fish without the involvement of an arthropod vector, although SAV can replicate in mosquito cells *in vitro* [4]. The mosquito-borne alphaviruses are generally subdivided based on their geographic origin. The New World alphaviruses include Venezuelan, Western, and Eastern equine encephalitis viruses (V/W/E-EEV) and are mostly associated with encephalitic disease in horses and humans. The Old World alphaviruses include the Sindbis virus (SINV) group, Barmah Forest virus (BFV), O’nyong’nyong virus (ONNV), Ross River virus (RRV), Semliki Forest virus (SFV), and chikungunya virus (CHIKV) and can cause symptoms of high fever, rash, incapacitating arthralgia, and chronic arthritis [5,6].

**Figure 1:**
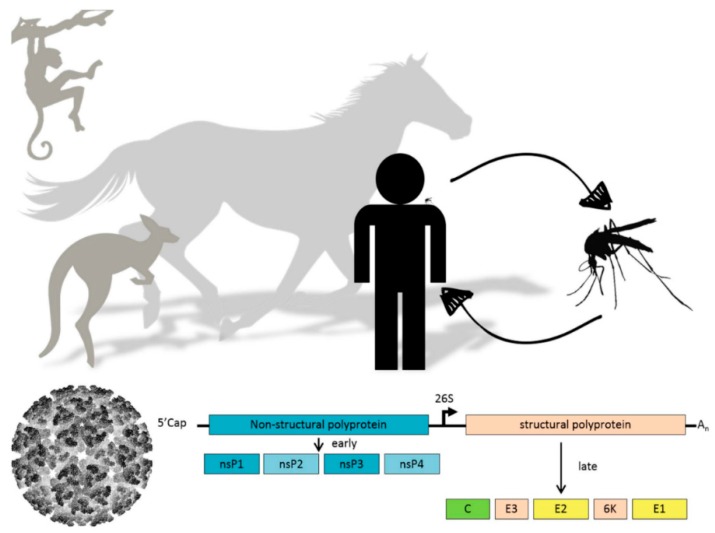
(**Top**) Schematic representation of alphavirus transmission cycle; (**Bottom**) Alphavirus particle and schematic representation of alphavirus genome organization. Translation of the nonstructural polyprotein (blue) can occur immediately from the positive-sense genomic RNA template, whereas the structural polyprotein (yellow) is expressed later in infection from a subgenomic messenger RNA template.

Alphaviruses can have a very diverse vertebrate and invertebrate host range [7]. CHIKV has evolved from a sylvatic cycle between forest dwelling mosquitoes and primates to a completely urbanized infection cycle in which urban *Aedes albopictus* and *Aedes aegypti* mosquitoes are the primary vectors and humans the primary hosts. A single amino acid change in one of the envelope glycoproteins substantially enhanced transmission by the Asian tiger mosquito *Aedes albopictus*, which sparked recent CHIKV outbreaks in Asia and Europe [8]. Other alphaviruses, e.g., RRV and BFV, are transmitted by various Australian mosquito species from different genera and their local reservoirs are macropods such as kangaroos and wallabies [9,10]. Each year, RRV and BFV are together responsible for thousands of human infections in Australia. Viruses from the SINV group use birds as their main amplifying hosts and in Scandinavia hundreds of human cases are reported annually (Figure 1) [11]. 

Since the discovery of CHIKV in 1952, sporadic CHIKV outbreaks have been recorded in central Africa and southern Asia [12]. However, from 2001 onwards several major outbreaks have occurred affecting the islands of Mauritius, Madagascar, Mayotte, and Reunion. On Reunion CHIKV affected up to a third of the human population during the outbreak of 2005/2006. In 2006, mainland India suffered a major outbreak, resulting in more than 1.4 million infected individuals. CHIKV activity has continued throughout Southern Asia where CHIKV is currently endemic [13]. In 2007, the first autochthonous transmission of CHIKV occurred on the European continent, infecting almost 250 people in Italy [14]. In 2010 and 2014, CHIKV was again transmitted on European territory in the southeast of France [15,16]. In October 2013, the first cases of autochthonous CHIKV transmission in the western hemisphere were detected in the French Caribbean [17]. Within the year, the distribution of CHIKV over the American continent had expanded, infecting over a million people across the Americas [18]. CHIKV is now co-circulating with dengue virus and Zika virus throughout South and Middle America and is likely to remain endemic for decades to come.

The alphavirus single-stranded, positive-sense RNA ((+)RNA) genome of approximately 11.8 kb is flanked by a 5’- and a 3’-untranslated region (UTR) and contains two open reading frames (ORFs). The 5’ ORF is directly translated from the genomic RNA into a polyprotein that contains the four nonstructural proteins (nsP) 1234, which is proteolytically cleaved into individual nsPs (Figure 1). The function of the individual nsPs in alphavirus replication has recently been reviewed in much detail [19]. Briefly, nsP1 is the membrane anchor of the replication complex (RC) and possesses guanine-7-methyltransferase (MTAse) and guanylyl transferase activities necessary for capping of the viral RNA [20,21]. NsP2 is the protease that cleaves the nonstructural polyprotein, has helicase activity and a methyltransferase-like domain [22]. The function of nsP3 in viral RNA replication is not understood, yet the protein is an essential component of the RC and is highly phosphorylated [23]. NsP4 is the RNA-dependent RNA polymerase [24,25], which is the first nsP to be proteolytically cleaved from the polyprotein. Together with the remaining nsP123 peptide, nsP4 forms a short-lived RC that produces the complementary negative-sense ((−)RNA). Further cleavage by the nsP2 protease results in four individual nsPs, which collectively form the RC that produces the (+)RNA genomes and subgenomic mRNAs [26]. The subgenomic mRNA is translated into the structural polyprotein. The viral capsid (C) protein is autocatalytically cleaved and encapsidates viral (+)RNA genomes in the cytoplasm to form nucleocapsids. The envelope proteins E1 and E2 mature while they are transported through the endoplasmic reticulum (ER) and Golgi apparatus to the plasma membrane, where they interact with the nucleocapsids and budding of progeny virus occurs [27]. Alphavirus replication is relatively fast, resulting in acute disease in vertebrate hosts and strong cytopathic effects in vertebrate cell culture. In contrast, infection of mosquito cells results in persistent infection and shows little to no sign of fitness loss in infected mosquitoes.

**Figure 2:**
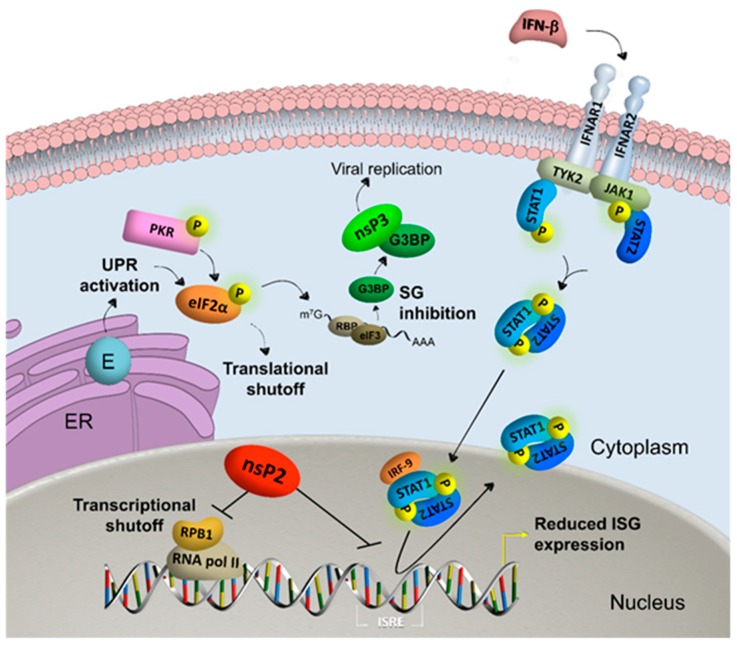
Alphavirus nonstructural protein 2 (nsP2) reduces interferon (IFN)-stimulated gene (ISG) expression by inducing transcriptional shutoff via RNA polymerase II (RNA pol II)-mediated host cell transcription by degradation of RNA polymerase subunit RPB1 and blocking the IFN-induced janus kinase-signal transducer and activator of transcription (JAK-STAT) pathway. Viral envelope (E) proteins activate the unfolded protein response (UPR) in the endoplasmic reticulum (ER) and together with kinases such as protein kinase R (PKR) phosphorylate eukaryotic translation initiation factor 2α (eIF2α). The subsequent induction of stress granules (SG) is inhibited by viral nonstructural protein 3 (nsP3), which sequesters Ras GTPase-activating protein-binding protein (G3BP) into viral granules that favor viral replication. General translational shutoff is additionally induced by alphavirus infection independent of eIF2α phosphorylation. Abbreviations: interferon-α/β receptor (IFNAR); tyrosine kinase (TYK); interferon regulatory factor (IRF); interferon stimulated response element (ISRE).

The recent outbreaks caused by CHIKV in Europe and the Americas illustrate that alphaviruses can adapt to new environments and different vector populations. Currently there are no vaccines or antivirals available for human use. A better understanding of the way alphaviruses modulate host cell antiviral and stress responses will further the field of antiviral research. To counter the array of vertebrate immune responses, alphaviruses have evolved to obstruct antiviral responses by inhibiting specific signaling pathways and by modulating general cellular processes, most importantly host cell shut-off caused by the inhibition of general cellular transcription and/or translation [24,28]. Ultimately, apoptotic cell death is the result of alphavirus infection, at least in vertebrate cells. The study of the interactions of alphaviral proteins with the autophagy machinery to delay apoptosis [29,30] is a developing research field that will not be covered here in detail. This review focuses on how Old World alphaviruses inhibit antiviral responses by modulating host cell transcriptional and translational processes and specific major antiviral and stress responses (Figure 2).

## 2. Transcriptional Shut-Off

Old World alphaviruses cause highly cytopathic infections in vertebrate cell culture systems. The identification of an attenuated SINV isolate and subsequent sequencing of the nonstructural genes revealed a single amino acid substitution in the C-terminal domain of SINV nsP2 (Proline to Serine, at amino acid position 726) that was able to establish a persistent infection in BHK cells [31,32]. Mutations in homologous sites of SFV [33,34] and CHIKV (P718) [35,36] resulted in attenuated and less cytopathic viral RNA replication, respectively. NsP2 translocates to the nucleus of infected cells, making use of a nuclear localization signal (NLS) that consists of a central lysine and arginine (e.g., CHIKV ^649^KR^650^) or multiple arginines (e.g., SFV ^649^RRR^651^) [35,37]. Mutations in the NLS of SFV nsP2 ^649^RDR^651^ almost completely blocked nuclear translocation of nsP2, but had no effect on SFV-induced cytopathicity or host cell shut-off [38,39]. Although this mutation did not result in a fully attenuated phenotype in BHK cells, it did reduce the viral neuropathology of mice [38,40]. A more recent study showed that different mutations in the NLS of SFV nsP2 (^649^DDR^651^ and ^649^RDD^651^) completely blocked nsP2 from entering the nucleus and reduced SFV-induced cell death [34]. The SFV nsP2 ^649^DDR^651^ and ^649^RDD^651^ mutations retained more nsP2 in the cytoplasm compared to the ^649^RDR^651^ variant [34], which may explain the contrasting results. Mutations in Old World alphavirus nsP2 that render the virus non-cytopathic, either in the NLS or P718 (P726 in SINV), all lose the ability to rapidly reduce host cell protein synthesis, even though nsP2 variants with P718 and P726 amino acid substitutions still translocate into the nucleus [34,35,41].

Once in the nucleus, nsP2 induces the degradation of RNA polymerase II subunit RPB1, which results in general host cell transcriptional shut-off and consequent cytopathic effects in mammalian cells [42]. A similar strategy is used by members of the *Picornaviridae* and *Bunyaviridae* families, which also enforce reduced host cell RNA transcription via the inhibition of RNA polymerase II [43,44,45,46,47,48]. A recent proteomics study showed that during CHIKV infection, most subunits of the RNA polymerase II complex were progressively degraded [49]. Degradation of RPB1 was shown to be induced by nsP2 proteins from SINV, SFV, and CHIKV and therefore seems to be conserved across Old World alphavirus species. The protease inhibitor MG132 was able to revert degradation of RPB1 by nsP2, suggesting that nsP2 utilizes cellular proteolytic processes for the degradation of RNA polymerase II. Using mutational analysis of nsP2, the degradation of RPB1 was attributed to a functional helicase domain and the C-terminal SAM-dependent MTAse-like domain, which is believed not to have MTAse activity [22]. Interestingly, the attenuating mutation in the C-terminal domain (SINV: P726G) was sufficient to abolish RPB1 degradation [42]. Taken together, this strongly suggests that cytopathicity and host shut-off are intrinsically linked.

Attenuating mutations in the proline of nsP2 (CHIKV: P718) not only reduce RPB1 degradation but also greatly affect viral RNA replication. Interestingly, a single attenuating mutation can be sufficient to establish persistent SINV or SFV, but not CHIKV infection in vertebrate cells. CHIKV requires mutations either at positions P718 and KR649 or P718 with a second site mutation (e.g., D711) to establish noncytopathic alphavirus RNA replication [35,36]. These mutations have differential effects on genomic and subgenomic alphavirus RNA production, suggesting that the cytopathicity induced by alphavirus replication, although clearly linked, does not solely depend on transcriptional shut-off but—at least in the case of CHIKV—requires heavily reduced viral replication as well [35]. New World alphaviruses also evolved to inhibit host mRNA transcription. However, it is not nsP2 but the viral capsid protein that inhibits cellular transcription [50]. By forming a complex with nuclear import and export factors it obstructs the nuclear pore and consequently nuclear trafficking, which has been suggested to account for the observed reduction in host mRNA transcription [51]. In mosquito cells alphavirus infection is less cytopathic and is not associated with degradation of the mosquito homologue of RPB1 [42]. This indicates that there is a need to shut down general translation as a means to inactivate the vertebrate antiviral immune system, but not in insect cells.

## 3. Translational Shut-Off

Alphaviruses genomes are flanked with a 5’ cap (N7mGppp) and a poly(A) tail at the 3’ end, which enables the direct translation of the nonstructural ORF [24]. A key factor in cellular translation is eukaryotic translation initiation factor 2 (eIF2), which controls translation as phosphorylation of the α subunit renders eIF2 unable to be recycled back into its active GTP-bound state, resulting in a general translational shut-off [52]. eIF2α is phosphorylated by four kinases that sense cellular stress. Protein kinase R (PKR) senses double-stranded RNA, PKR-like ER kinase (PERK) senses unfolded proteins in the ER, whereas GCN2 and HRI are activated by nutrient starvation and heme deficiency, respectively [52].

Double-stranded RNA is an intermediate of RNA virus replication. Translational shut-off via dsRNA recognition by PKR can effectively block viral replication [53,54,55]. Most viruses therefore prevent the activation of PKR and/or subsequent phosphorylation of eIF2α to allow and promote the translation of viral proteins [56,57,58,59]. Although inhibited phosphorylation of eIF2α by CHIKV nsP4 has been reported [60], it is well established that alphaviruses allow the phosphorylation of eIF2α during infection [28,61,62,63,64]. However, the translation of alphaviral structural proteins from their subgenomic messenger is unaffected by the phosphorylation of eIF2α. A stable RNA hairpin loop structure in the 26S promoter of the subgenomic mRNA from SINV and SFV stalls the ribosome on the correct AUG, providing resistance to eIF2α phosphorylation and thereby enhancing translation of the viral subgenomic mRNA [62,65,66,67]. Details of this translation initiation mechanism have become clearer through RNA structure modeling of the alphavirus hairpin onto the 40S ribosomal subunit [68]. Although for some alphaviruses, including CHIKV, such a RNA structure in the 26S promotor could not be determined, eIF2α phosphorylation early in infection does not prevent efficient translation of structural subgenomic mRNA [63,64].

It is interesting to note that the general host cell translational shut-off during alphavirus infection has been shown to be independent of PKR and eIF2α phosphorylation, indicative of an additional mechanism by which alphaviruses modulate the translational machinery of the host [28,63]. Indeed, SFV infection has been shown to reduce levels of phosphorylated eIF4E, which is the cap-binding protein within the eIF4F complex [69]. It has also been suggested that alphavirus nsP2 proteins may alter ribosomes by association with ribosomal protein S6 of vertebrates and mosquitoes [70], but the mechanistic details are not entirely clear. Mutations in the C-terminus of nsP2 that inhibited cleavage between nsP2 and nsP3 were shown to inhibit translation of cellular mRNAs, but not transcriptional shut-off [71]. The underlying mechanism of translational shut-off remains elusive, however, and needs further investigation. Be it at the level of transcription or translation, shut-off of host cell protein synthesis not only affects cellular processes and previously mentioned viral cytopathicity, but most likely evolved foremost as a way to inhibit antiviral responses.

## 4. Interferon Response

The IFN response is arguably the most potent innate antiviral response that vertebrates possess [72]. Pattern recognition receptors (PRRs) recognize viral elements, resulting in the expression and secretion of IFNs. IFNs activate neighboring cells via transmembrane receptors (IFNAR), which signal through the janus kinase-signal transducer and activator of transcription (JAK-STAT) pathway. STAT1/2 dimers are activated by phosphorylation and translocate to the nucleus, resulting in the upregulation of IFN-stimulated (antiviral) genes (ISG). Inhibition of the IFN response is absolutely essential for alphaviruses to establish productive infections in mammalian hosts [73,74,75]. General host cell shut-off, induced by SINV nsP2, affects the expression of IFNs and ISGs [41,71]. In addition, alphavirus infection is resistant to IFNs *in vitro* and *in vivo*, as viral titers and replicon RNA replication were unaffected and ISG expression was still inhibited post-IFN treatment. However, when treated with IFNs prior to infection, ISGs were readily expressed and virus replication was severely reduced [73,76].

Interestingly, IFNs did not induce the nuclear translocation of STAT dimers when cells were infected with CHIKV [76] or VEEV [77]. The absence of nuclear phosphorylated STAT (pSTAT) in CHIKV infected cells was independent of general host shut-off as cellular levels of STAT1 were unaffected by CHIKV RNA replication and complete inhibition of protein synthesis with cycloheximide did not affect STAT nuclear translocation during the course of the experiments. Transient expression of individual CHIKV nsPs revealed that nsP2 effectively inhibited JAK-STAT signaling [76]. Furthermore, the P718 mutation in the C-terminal domain of nsP2 that abolished cytopathicity and transcriptional shut-off still inhibited the nuclear localization of pSTAT1 during transient expression [35]. In contrast, mutations in the NLS of nsP2 (KR649AA) completely reinstated the JAK-STAT signaling pathway, indicating that the nuclear localization of nsP2, but not the ability of nsP2 to cause transcriptional shut-off, is essential for the inhibition of STAT1 nuclear localization [35,76]. Determination of the relative contribution of suppressing ISG expression via host shut-off or the direct inhibition of JAK-STAT signaling needs further experimentation, but during infections in vertebrates these may well complement each other and be necessary for establishing and sustaining a transmissible infection, given that even with all these viral countermeasures the IFN response is still regarded as the main driver behind viral clearance [78].

In contrast to infection in the vertebrate host, arboviruses persistently infect their arthropod vector with little to no pathogenesis and without detrimental effects on vector fitness parameters. RNA interference (RNAi) is the most potent antiviral mechanism that the virus has to overcome to be transmitted [79]. The general consensus in the field is that RNAi largely controls infection at the level of the mosquito midgut. However, ONNV virus infection in *Anopheles gambiae* showed that RNAi limits virus dissemination beyond the midgut [80]. So far, alphavirus-encoded RNAi suppressor proteins have not been identified despite the active antiviral RNAi response. For SFV, virus-derived siRNAs generated from “hot-spot” regions were less efficient at silencing than “cold-spot”-derived siRNAs, suggesting that low-activity siRNAs may be generated as abundant RNA decoys to suppress antiviral RNAi [81]. Insects also possess additional immune pathways, some of which are analogous to those found in humans and other vertebrates, including the JAK-STAT signaling pathway. *In vitro* experiments showed that SFV but not CHIKV infection affects JAK-STAT signaling in *Aedes aegypti* cells [82,83]. Future work will elucidate the relative importance of these antiviral pathways in controlling alphavirus infection and will shed light on the mechanisms employed by alphaviruses to modulate antiviral response in the arthropod vector.

## 5. Unfolded Protein Response

The unfolded protein response (UPR) is activated by ER stress, which generally occurs when unfolded or misfolded proteins are present in the ER. The expression of large amounts of viral glycoproteins that are post-translationally modified in the ER can lead to ER stress and trigger the UPR [52,84]. Recent insights suggest that the UPR can support important antiviral responses [85]. Glycoproteins from alphaviruses SFV and CHIKV have the potential to activate the UPR [64,86]. This results in the induction of several key components of the UPR. DsRNA-dependent PERK is an ER transmembrane kinase that upon sensing unfolded proteins in the ER phosphorylates eIF2α, which is activated early during SINV and CHIKV infections [60,61]. Under conditions of ER stress, eIF2α phosphorylation, and subsequent translational repression of general protein expression, the expression of stress-related proteins (e.g., ATF4) is upregulated. Additionally, upon sensing unfolded proteins in the ER, the mRNA of transcription factor XBP1 is spliced, resulting in an active UPR transcription factor. XBP1 mRNA splicing is initiated during SFV infection [86]. We demonstrated that, although eIF2α is phosphorylated and part of the XBP1 mRNA pool is spliced, XBP1 is not present in the nucleus and the upregulation of ATF4 and UPR target genes is completely inhibited in CHIKV-infected cells. This suggests an eIF2α-independent block in translation [64].

Transient expression studies of nsPs demonstrated that nsP2 is responsible for preventing an effective UPR and that point mutations in nsP2 that render the protein non-cytopathic by eliminating its function in host shut-off (KR649AA or P718S) reversed the nsP2-mediated inhibition of the UPR [64]. Perhaps the failure to constrain the UPR contributes to the attenuated phenotype seen for alphaviruses carrying these mutations [32,34]. We postulate that the host cell shut-off, which is governed by CHIKV nsP2, is responsible for the inhibition of the UPR, not by preventing activation of ER sensors but mainly by preventing the upregulated expression of ATF4, active XBP1, and additional UPR target genes. Recent research has shown that CHIKV triggers both apoptosis and autophagy via the independent induction of ER stress and oxidative stress pathways [87]. Ultimately, apoptotic cell death is delayed by inducing the IRE1α-XBP-1 pathway in conjunction with ROS-mediated mTOR inhibition, suggesting that autophagy may limit CHIKV pathogenesis.

## 6. Stress Granules

In mammalian cells, translational attenuation via the phosphorylation of eIF2α results in the formation of stress granules (SG). SGs contain mRNPs and stalled translation initiation complexes, and often form during viral infections. In SFV infection, TIA-1/R positive granules are formed transiently, but later they are disassembled with ongoing viral replication. Many viruses effectively counteract the assembly of SGs, suggesting their involvement in antiviral activity (reviewed in [88]). In fact, a number of antiviral RNA binding proteins localize to SG, e.g., retinoic acid-inducible gene I (RIG-I) and melanoma differentiation-associated gene 5 (MDA5) [89,90,91]. These RIG-I-like receptors have been recognized as some of the most potent antiviral cytoplasmic PRRs [92,93]. Recently, a specific interaction between the PxxP domain of SG protein Ras-GTPase activating SH3-domain binding protein (G3BP) and PKR was shown to activate PKR, indicative of a strong antiviral role of SG and G3BP [94]. Together this suggests that the formation of SGs is intertwined with other cellular innate antiviral responses. Thus, considering the growing evidence that SGs augment innate antiviral responses, it may not come as a complete surprise that alphaviruses have evolved ways to inhibit SG formation.

Old World alphavirus nsP3 interacts with vertebrate G3BP and the mosquito homologue Rasputin (Rin) in mammalian and mosquito cells, respectively [95,96,97]. In both invertebrate and vertebrate cells nsP3 forms cytoplasmic granules with Rin/G3BP. The cytoplasmic granules in vertebrates are clearly distinct from bona fide SG as they lack other SG components and do not respond to chemical stimulations that either induce (arsenite) or disassemble (cycloheximide) normal SGs [97]. As a consequence of the sequestration of G3BP into these viral nsP3-G3BP granules, the assembly of bona fide SG is effectively inhibited [97,98]. The C-terminus of Old World alphavirus nsP3 has a high degree of variability between alphavirus species, but does contain conserved peptide motifs. In both insect and mammalian cells, Rin and G3BP are sequestered into nsP3-granules via an interaction between the conserved N-terminal NTF2-like domain of Rin/G3BP and two conserved FGDF repeats in the C-terminal variable domain of Old World alphavirus nsP3 that resemble the amino acid residues normally bound by NTF2-like domains [96,99,100].

The importance of the nsP3–G3BP interaction became apparent in a deletion mutagenesis study with SFV. Deletion of the C-terminal 30 amino acids, which contain the G3BP binding domains [99], severely reduced SFV replication [101], strengthening the hypothesis that the interaction between nsP3 and Rin/G3BP has a positive effect on viral replication. We showed that deletion of a conserved SH3 domain-binding motif in the nsP3 C-terminus, just upstream of the two FGDF repeats, proved to be lethal for CHIKV replication and disrupted the granular co-localization of nsP3 with G3BP [97].

The exact mechanism by which Rin/G3BP enhances viral replication in both vertebrates and invertebrates is still unknown; however, G3BPs are not directly involved in the translation of incoming genomes, nor in genome replication. Perhaps G3BPs support the switch between viral nonstructural polyprotein translation and negative strand RNA replication [102]. Transcriptional silencing of G3BP1 and G3BP2 in mammalian cell culture decreased levels of CHIKV RNA replication, CHIKV protein expression, and progeny virus titers [102]. Similarly, SINV replication was also negatively affected by the simultaneous depletion of G3BP1/2 [102,103,104]. In contrast, Rin depletion in mosquito cells *in vitro* did not alter levels of CHIKV replication and/or protein expression, suggestion that important differences between vertebrates and invertebrates may exist. Depletion of Rin in live *Aedes albopictus* mosquitoes, however, significantly reduced CHIKV infection rates, again highlighting that the interaction between nsP3 and Rin elicits proviral effects *in vivo* [96].

In Drosophila, Rin has been suggested to form RNase inhibitor complexes [105], which could protect CHIKV RNA replication during the initial infection of the mosquito, e.g., in the midgut. SGs are triage centers of RNA binding proteins and AGO2 has also been found to localize to SGs [106,107]. It is tempting to speculate that arboviruses modulate the SG response in invertebrates to decrease but not completely abolish the effects of RNAi and possible other antiviral pathways to balance appropriate levels of viral replication and fitness of the vector. Clearly, this could be a focus area for further research. These observations may also propose that small molecule modulators of the stress response find an application in antiviral treatments by stimulating antiviral responses, but perhaps more effectively by reducing viral replication rates [100]. The interaction between nsP3 and Rin/G3BP can provide a novel target to interfere with the alphavirus transmission cycle and/or the disease associated with alphavirus infection.

## 7. Conclusions

Most of the work summarized in this review is based on the model alphaviruses SINV, SFV, and, to a lesser extent, CHIKV. While all alphaviruses fall within a single genus in the *Togaviridae* family, the diversity is large, not only with respect to host and vector range, but also with respect to their persistence in arthropods and their pathogenesis in aquatic and terrestrial vertebrates. It therefore remains to be seen how universally applicable the alphavirus dogmas remain across all the different members of the Alphavirus genus. It is clear that more and more differences in alphavirus–host interactions will be uncovered in the future as our understanding of this group of viruses becomes more detailed. Nevertheless, despite an increase in knowledge over the past decades, we are still far away from effectively combating the most important alphavirus diseases.

Currently there are no vaccines or antiviral compounds available for human use that are effective against any of the Old World alphavirus infections, but preclinical research shows that several compounds may have potential for clinical testing [108,109,110,111]. These viruses are, however, readily cleared by an operational vertebrate innate immune response *in vivo*, which produces large amounts of IFNs [78]. When IFNs are administered prior to (or very early during) infection they do effectively protect against disease in experimental animal models [73,75]. IFNs are regularly used as antiviral treatment during chronic viral infections; however, they lack effectiveness when used to treat acute viral infections (e.g., alphaviruses) [112]. Host cell shut-off established early in infection combined with specific nsP2-mediated inhibition of JAK-STAT signaling clearly provides a rationale for the ineffectiveness of IFN treatment in response to clinical alphavirus infections [35,42,76].

The UPR has been proposed to strengthen and specify the IFN response [85]. This explains why CHIKV induced host shut-off has evolved to also inhibit UPR. Similar to the use of IFNs, the replication of several RNA viruses, including SINV, was inhibited when the UPR was activated with a small molecule deubiquitinase inhibitor prior to infection [113,114]. However, activating the UPR may be more difficult when host cell shut-off is established [64]. Taken together, this suggests that attempts to activate antiviral pathways that are crippled as a result of either host cell shut-off or specific inhibition in response to alphavirus infection are futile.

Alternatively, compounds that directly interfere with other stress signaling pathways, viral replication, virion assembly, or virus entry may be more effective. The interaction between CHIKV nsP3 and G3BP/Rin inhibits the stress granule response, but also exerts a positive effect on viral replication both in vertebrate cells and the mosquito vector [96,97,102]. Compounds that bind the disordered C-terminal hyper variable domain of nsP3 with high affinity and disturb the interaction between G3BP and nsP3 may have the potential to increase the antiviral response by re-establishing a functional SG response, while at the same time reducing viral replication rates. Homologous interactions between nsP3 and mosquito Rin may also provide a novel target to interfere with the CHIKV transmission cycle [97].

Finally, it is clear that alphaviruses have evolved efficient ways of preventing or inhibiting antiviral responses in vertebrates to maximize virus replication during a rapid and lytic infection (summarized in Figure 2). Activation of antiviral pathways that are affected by host cell shut-off seem unlikely to be effective, pointing to the need for compounds that abolish host shut-off or directly inhibit viral replication.
